# Poverty, Disease, and the Ecology of Complex Systems

**DOI:** 10.1371/journal.pbio.1001827

**Published:** 2014-04-01

**Authors:** Calistus N. Ngonghala, Mateusz M. Pluciński, Megan B. Murray, Paul E. Farmer, Christopher B. Barrett, Donald C. Keenan, Matthew H. Bonds

**Affiliations:** 1 Department of Global Health and Social Medicine, Harvard Medical School, Boston, Massachusetts, United States of America; 2 National Institute for Mathematical and Biological Synthesis (NIMBioS), The University of Tennessee, Knoxville, Tennessee, United States of America; 3 Department of Environmental Science, Policy and Management, University of California, Berkeley, Berkeley, California, United States of America; 4 Dyson School of Applied Economics and Management and Department of Economics, Cornell University, Ithaca, New York, United States of America; 5 Université de Cergy-Pontoise et Théorie Economique, Modélisation, Application (THEMA), Cergy-Pontoise, France; 6 PIVOT, Boston, Massachusetts, United States of America

## Abstract

Understanding why some human populations remain persistently poor remains a significant challenge for both the social and natural sciences. The extremely poor are generally reliant on their immediate natural resource base for subsistence and suffer high rates of mortality due to parasitic and infectious diseases. Economists have developed a range of models to explain persistent poverty, often characterized as poverty traps, but these rarely account for complex biophysical processes. In this Essay, we argue that by coupling insights from ecology and economics, we can begin to model and understand the complex dynamics that underlie the generation and maintenance of poverty traps, which can then be used to inform analyses and possible intervention policies. To illustrate the utility of this approach, we present a simple coupled model of infectious diseases and economic growth, where poverty traps emerge from nonlinear relationships determined by the number of pathogens in the system. These nonlinearities are comparable to those often incorporated into poverty trap models in the economics literature, but, importantly, here the mechanism is anchored in core ecological principles. Coupled models of this sort could be usefully developed in many economically important biophysical systems—such as agriculture, fisheries, nutrition, and land use change—to serve as foundations for deeper explorations of how fundamental ecological processes influence structural poverty and economic development.

## Abstract 2

Coupled models of ecology and economic growth can provide key insights into the formation of poverty traps that arise from complex interactions between biosocial and biophysical processes.

## Introduction

In his landmark treatise, *An Essay on the Principle of Population* [1], Reverend Thomas Robert Malthus argued that population growth will necessarily exceed the growth rate of the means of subsistence, making poverty inevitable.The system of feedbacks that Malthus posited creates a situation similar to what social scientists now term a “poverty trap”: i.e., a self-reinforcing mechanism that causes poverty to persist [2],[3]. Malthus's erroneous assumptions, which did not account for rapid technological progress, rendered his core prediction wrong: the world has enjoyed unprecedented economic development in the ensuing two centuries due to technology-driven productivity growth. Nonetheless, for the billion people who still languish in chronic extreme poverty, Malthus's ideas about the importance of biophysical and biosocial feedback (e.g., interactions between human behavior and resource availability) to the dynamics of economic systems still ring true. Indeed, while they were based on observations of human populations, Malthus ideas had reverberations throughout the life sciences. His insights were based on important underlying processes that provided inspiration to both Darwin and Wallace as they independently derived the theory of evolution by natural selection. Likewise, these principles underlie standard models of population biology, including logistic population growth models [4], predator-prey models [5],[6], and the epidemiology of host-pathogen dynamics [7].

The economics literature on poverty traps, where extreme poverty of some populations persists alongside economic prosperity among others, has a history in various schools of thought. The most Malthusian of models were advanced by Leibenstein [8] and Nelson [9], who argued that interactions between economic, capital, and population growth can create a subsistence-level equilibrium (see Box 1). Today, the most common models of poverty traps are rooted in neoclassical growth theory, which is the dominant foundational framework for modeling economic growth. Though sometimes controversial, poverty trap concepts have been integral to some of the most sweeping efforts to catalyze economic development, such as those manifest in the Millennium Development Goals [10]. The modern economics literature on poverty traps, however, is strikingly silent about the role of feedbacks from biophysical and biosocial processes. Two overwhelming characteristics of under-developed economies and the poorest, mostly rural, subpopulations in those countries are (i) the dominant role of resource-dependent primary production—from soils, fisheries, forests, and wildlife—as the root source of income [11],[12], and (ii) the high rates of morbidity and mortality due to parasitic and infectious diseases [13]. For basic subsistence, the extremely poor rely on human capital that is directly generated from their ability to obtain resources, and thus critically influenced by climate and soil that determine the success of food production. These resources in turn influence the nutrition and health of individuals, but can also be influenced by a variety of other biophysical processes. For example, infectious and parasitic diseases effectively steal human resources for their own survival and transmission. Yet scientists rarely integrate even the most rudimentary frameworks for understanding these ecological processes into models of economic growth and poverty.

**Box 1. Economic Growth Models and Poverty Traps**

The most common models of poverty traps can be coarsely grouped into two schools of thought. One school, a macro-oriented approach known as neoclassical growth theory, has generally viewed economic growth as an inevitable outcome of normal market processes driven by savings and technological progress that dictate patterns of capital accumulation. These patterns were thought to lead to convergence over time in economic growth rates across countries [36],[75]. When and where the convergence hypothesis failed, long-term economic stagnation was accordingly attributed to factors that hindered the proper functioning of market processes, such as weak government and economic institutions [76],[77]. More recent variants of growth theory endogenize rates of savings and technological change, emphasizing the role of reinforcing economic feedback and other positive spillover effects of investment that influence equilibrium rates of productivity growth [77]–[79]. Though not a primary focus of this literature, Solow's original paper presented a model of a poverty trap based on feedbacks with population growth. Today, the most common poverty trap models are rooted in the neoclassical model [2].

The second school, advanced mainly by more micro-oriented development economists, emphasizes the potential for poverty traps formed by feedbacks between income, capital accumulation, population, or occupational choice, where long-term outcomes depend on initial conditions in systems characterized by multiple equilibria [2],[3],[80]–[82]. Foundational work in this literature advanced by Nelson [9] and Leibenstein [83] had substantial Malthusian overtones, where feedbacks between population and resource availability were key drivers. Over time, more explicit nonlinear biological mechanisms have been posited, such as the relationships between nutrition and labor productivity at the individual level [61],[63],[84]. In these models, optimal economic behaviors for initially poor subpopulations differ from those of initially better-off neighbors, leading to conditional divergence in standards of living that even endogenous growth theory cannot convincingly explain. Such arguments, along with complementary macro-oriented theories, have been integral to the “Big Push” efforts manifest in the Millennium Development Goals [10]. These two schools of thought are not necessarily mutually exclusive: like endogeneous growth theory, many micro-oriented poverty trap models trace their roots back to neoclassical growth theory [33],[85],[86].

This gap in the literature represents a major missed opportunity to advance our understanding of coupled ecological-economic systems. Through feedbacks between lower-level localized behavior and the higher-level processes that they drive, ecological systems are known to demonstrate complex emergent properties that can be sensitive to initial conditions [14],[15]. A large range of ecological systems—as revealed in processes like desertification, soil degradation, coral reef bleaching, and epidemic disease—have been characterized by multiple stable states, with direct consequences for the livelihoods of the poor [16]–[22]. These multiple stable states, which arise from nonlinear positive feedbacks, imply sensitivity to initial conditions. While Malthus's original arguments about the relationship between population growth and resource availability was simplistic (resulting in only one stable state of subsistence poverty), they led to more sophisticated characterizations of complex ecological processes. In this light, we suggest that breakthroughs in understanding poverty can still benefit from two of his enduring contributions to science: (i) models that are true to underlying mechanisms can lead to critical insights, particularly of complex emergent properties, that are not possible from pure phenomenological models; and (ii) there are significant implications for models that connect human economic behavior to biological constraints.

Here, we present a simple model of economic growth where capital accumulation is tied mechanistically to ecological processes. The framework is meant to be general enough to be adapted to a range of biosocial systems that are poised to be integrated with theoretical ecology. Relevant systems include biological symbionts of humans, such as those that provide essential resources to the poor (through, for example, agriculture, timber, fishing, and hunting), as well as those that remove those resources; i.e., natural enemies of humans and their symbionts (livestock predators, crop raiders, agricultural pests, termites, and infectious diseases of humans). For heuristic purposes, the model that we develop here focuses on human infectious diseases for the following reasons: (i) models of infectious diseases are contributing to major advances in ecological theory in general, with lessons that are applied to similar dynamic living systems such as fisheries, wildlife, and food production (in some cases, their effects on nutrition are clinically equivalent to low food intake [23]–[25]); (ii) infectious diseases are the leading killers of the poor and have been dominant natural enemies of humans throughout history [26]–[28]; and (iii) there is an emerging theoretical ecology literature that explicitly models the role of infectious diseases on poverty traps [29]–[32].

Both mathematical models of infectious diseases and of economic growth are based on dynamical systems that are canonical within their respective disciplines [7],[33]–[35]. Linking them is conceptually appropriate and methodologically straightforward. We show that the structure of these coupled systems can create bistable outcomes, and therefore traps in poverty and disease, depending on the number of pathogens in the system. We intentionally work with simple models, not to discount complexity, but to illustrate complex outcomes as emergent properties of parsimonious integrated models. This is not an empirical contribution and these models should not be confused for evidence. Instead, this is a conceptual framework to encourage the scientific community to contribute towards a more unified understanding of poverty. Developing and testing poverty trap models requires a breadth of scientific methods that reaches beyond the conventional domain of economics and draws on the ecology of complex systems.

## Economic Growth Model

The standard neoclassical economic growth model is a dynamic description of changes in capital over time [33],[34],[36]. In the original formulations, the term “capital” was meant to represent the physical inputs (such as infrastructure and equipment) that, when combined with labor, are used in the production of economic goods. Other forms of capital have become routinely incorporated into economic growth models, notably “human capital,” which commonly represents education and training of the workforce, but which can also represent health status [37]–[39]. The production process converts capital to output (and thus income), some of which is consumed and some of which is saved and reinvested. The processes of continually saving and reinvesting a portion of the total output into forming new capital is the source of capital accumulation and the basis of economic growth in the canonical framework.

**Figure 1:**
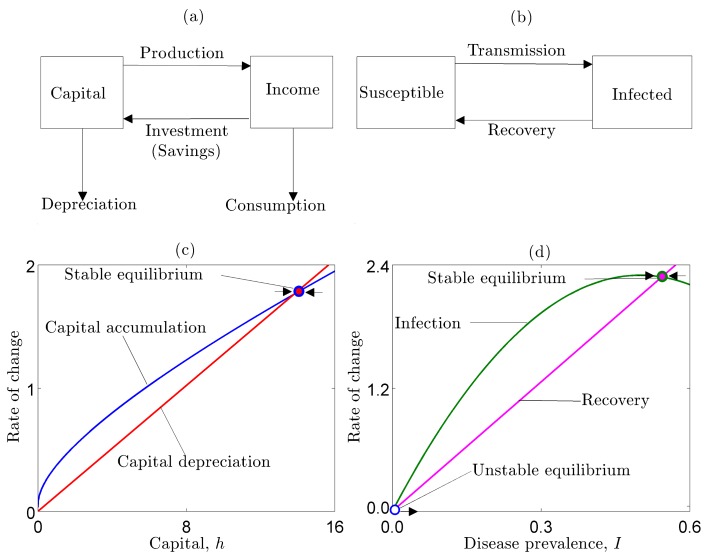
Schematics of (a) the neoclassical growth model, and (b) the SIS epidemic model demonstrate basic similarities in their structure. In the neoclassical growth model (c), the blue line represents capital accumulation and the red line represents capital depreciation. The steady state level of capital occurs where these curves intersect. In the typical epidemic model (d), the green line represents transmission (i.e., the force of infection) and the pink line represents recovery. The steady state prevalence of disease is where these curves intersect.

Figure 1a presents a schematic of the neoclassical growth model, which is illustrated graphically in Figure 1c (the corresponding mathematical models are provided in Text S1). The red line represents depreciation and the blue line represents the rate of accumulation (savings). Because of “diminishing returns,” the growth rate is relatively high at low levels of capital and then falls as capital accumulates. When the rate of savings equals the rate of depreciation, the system has reached equilibrium. The simplest model thus suggests that poor countries should grow faster than rich countries (“conditional convergence”), a prediction that came to define a substantial part of the early economic growth literature [36].

Though intentionally simplified, Figure 1c represents the textbook economic growth model based on the original Solow-Swan formulation, which continues to be the foundation of virtually all current models of economic growth. It is the point of departure for canonical poverty trap models in the economics literature [2],[33],[36]. As with foundational models in population biology, this original model has become modified in any of a host of useful directions, especially to explain empirical anomalies, such as divergence, where the gap between rich and poor countries expands over time (Box 1) [40]–[43].

**Figure 2:**
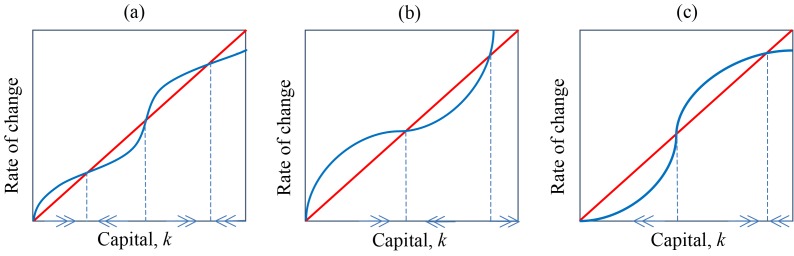
(a–c) are examples of nonlinear production functions extracted from the economics literature [2]. The x-axis is the stock of capital. The blue line represents the rate of capital accumulation (i.e., savings) and the red line represents that rate of capital depreciation. Income (generated from capital) will necessarily fall when the red line is above the blue line, and will rise when the reverse is true. (c) is the canonical depiction of a poverty trap, but (a–c) all have stable equilibria in the basin of attraction of a poverty trap, and unstable equilibria that represent a critical threshold of capital necessary for growth. These models are speculative, based on hypothetical scenarios, but are useful for demonstrating a range of theoretical possibilities. The scientific community should contribute to our understanding of how such nonlinearities might emerge from, or be nested within, real world biophysical systems.

In the standard poverty trap model, the production function exhibits nonlinear returns to investment, such that the rate of capital appreciation falls below capital depreciation in the early stages. This can occur, for example, when the rate of savings becomes very low due to low levels of capital (e.g., because the poor are unable to save), only to rapidly increase as wealth rises from these low levels, before finally experiencing diminishing returns. Because the shape of the production function has significant implications, the economics literature has explored a large range of them, a few nonlinear examples of which are presented in Figure 2. As phenomenological models that are not necessarily based on mechanistic understanding, they are often speculative. There is a critical opportunity for the scientific community to anchor these concepts with a scientific understanding of underlying biophysical drivers.

## Infectious Disease Model

Explicitly modeling the population ecology of infectious diseases can inform our understanding of the structure of feedbacks between human health and economic growth, can lead to more predictive and testable frameworks, and can ultimately allow for the exploration of potential unpredictable emergent properties of such complex systems. The classic 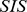 compartmentalized epidemiological model [35],[44]–[46] apportions the population into susceptible and infectious individuals (denoted by 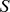 and 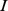, respectively). This is a typical population ecology model derived from predator-prey dynamics. In its general form, it is directly comparable to models of other kinds of biological systems that are relevant to the poor, such as the dynamics of fisheries, terrestrial wildlife, livestock, and agriculture (see Box 2) [47].

**Box 2. Population Ecology Models and Poverty Traps**

Infectious disease models stem from a long tradition of models in population biology that have been similarly developed for other living systems, with important implications for the livelihoods of the poor. One of the most basic kinds of systems is food. To demonstrate how the general framework that we use for infectious diseases can be applied to these other systems, we present two examples of food-related poverty trap models (Figure 4).

(a) The phase plot depicts the changes over time that occur at each combination of disease and human capital in the state space. Three equilibrium states are depicted: two stable equilibria (solid circles), and one unstable equilibrium (open circle) in between. Sample trajectories in the basin of attraction of the good equilibrium are denoted by blue lines, while sample trajectories in the basin of attraction of the poverty trap are denoted by red lines. Because the dynamics of infectious diseases occur at a more rapid time scale than the economic dynamics (which occur over the course of generations), we can assume that the disease equilibrates instantaneously; this two dimensional system then collapses to a one-dimensional system as depicted in Figure 2. The one-dimensional depiction is directly analogous to the canonical poverty trap models in the economics literature. (b) As a representative model from the ecology literature of an agricultural type of process, one can use models of plant growth, comparable to other forms of renewable resource models [18],[87],[88]. As with infectious diseases, these models depict changes over time of the state variable (plant density, 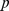), which is a function of key resources, such as water or soil nutrients. One then includes capital as a limiting factor for food production using a traditional production function (i.e., agricultural productivity depends on capital), which couples the system to economics (for details see Text S1). (c) Nutrition uptake can be modeled as a classic ecological consumer-resource system, such as has been used to represent rates of feeding and energy transfer [89]–[92]. The rate of nutrition uptake occurs according to a Holling functional response, and directly affects the rate of human capital acquisition, similar to the disease model (for details see Text S1). Because nutrition uptake is known to respond nonlinearly to resource availability, poverty traps can emerge [61].

The SIS model has specifically been used to study many diseases that serially re-infect their hosts and that do not confer permanent immunity, such as many vector-borne, sexually transmitted, parasitic and bacterial infections [46]. A large range of extensions of this model have been used to account for various epidemiologically important factors, such as demography, immunity, and seasonality, along with different forms of transmission, depending on the system and questions of interest [48]. As in the economic growth model, for clarity we focus on the simplest version of the system, a schematic of which is presented in Figure 1b and illustrated graphically in Figure 1d (explicit equations are provided in Text S1).

Notice from Figure 1 that the simplest disease and economic growth models share the same basic structure. Key parameters in the disease system are the transmission (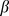) and recovery (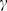) rates, which comprise the basic reproductive ratio, 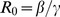. 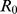 represents the average number of secondary infections in a totally susceptible population caused by a single infectious individual over the lifetime of the infection [46],[49]. The disease can persist endemically if 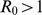. Thus, like the simple economic growth model, this epidemic model admits a maximum of one stable equilibrium: 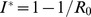, if 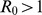.

## Coupled Disease-Economic Growth Model

While economic growth and infectious diseases are typically modeled as independent systems, in reality they are often highly coupled [30],[50],[51]. There is overwhelming evidence that economic and social conditions are major risk factors for infectious diseases [52],[53]. For example, transmission of diarrheal diseases and helminth infections can be prevented with well-made latrines or septic systems, each highly dependent on income. Similarly, clothes, shoes, clean water, screens, and bed nets, all reduce disease transmission but are often not available to the poor. In addition to prevention, income can affect disease by increasing recovery rates through both individual-level biological factors like nutrition-related immune responses, and health system responses, such as medical treatment. What all of these mechanisms have in common is that basic methods for preventing and treating disease require resources that are less available to the poor. By lowering the numerator (transmission) and increasing the denominator (recovery) of the basic reproductive ratio, income lowers the prevalence of disease in the population. Simple models drawn from the biology literature of how income influences these disease parameters have been presented in a recent series of papers [29]–[32] and are graphically presented in Text S1.

The evidence that disease impacts economic growth is also overwhelming [51],[54]–[60]. First, disease has obvious and direct effects on labor productivity by reducing ability to work [61]–[63]. This is a dominant consequence for chronic infections such as tuberculosis and HIV, but also important for infections that cause more temporary disability. There are also a wide range of more long-term influences on economic productivity from the effects of child disease on cognitive development and educational performance, which are fundamentally important to income-generation [37]. This has been especially identified for intestinal parasites (which cause anemia and iron depletion) and malaria [64]–[66]. In addition, there are the more subtle effects of survival rates on household decisions, such as reproductive behavior and long-term investments in education [59]. Because human capital is a significant mechanism through which the disease burden influences economic growth, we focus on human capital, 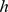, as a basis for model integration. We further generalize this model to incorporate 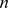 infectious diseases because the most important effects of health on economic growth occur through the cumulative effects of all infections.

**Figure 3:**
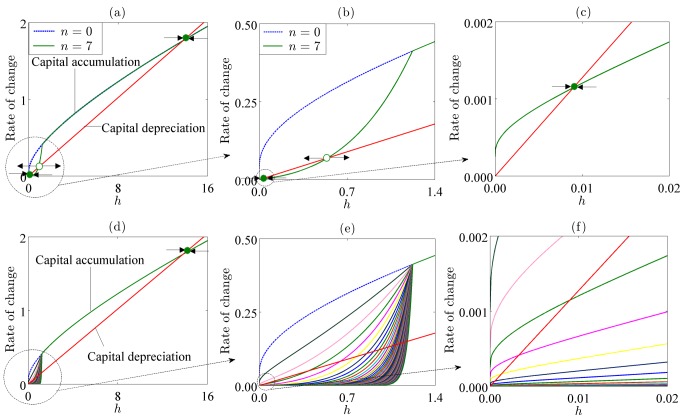
Multiple infections cause the appearance and expansion of the basin of attraction of poverty traps. For graphs (a) and (b) 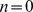 (dashed blue line) and 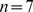 (solid dark green line). Graph (b) is a magnified version of the initial portion of graph (a), while graph (c) is a magnified version of the initial portion of graph (b) showing a stable positive poverty trap. The filled circles denote stable equilibria while the open circle denotes an unstable equilibrium. Graph (e) is a magnified version of the initial portion of graph (d), while graph (f) is a magnified version of the initial portion of graph (e). Each curve in graphs (d–f) represents the structure of capital accumulation for different numbers of pathogens.

Our modeling approach here builds on the work of [29]–[32], where explicit disease models are coupled to ad hoc economic models. Here, we explicitly link the disease models to economic growth theory, which allows for the ecological system to become explicitly comparable to canonical poverty trap models. In contrast to the uncoupled models, Figure 3 illustrates the effects of multiple diseases on the structure of human capital accumulation in this coupled system. The parameters are drawn from the literature where possible, and were otherwise calibrated from raw data on per capita income and the burden of infectious diseases (DALYs) for countries around the world (see Text S1).

**Figure 4:**
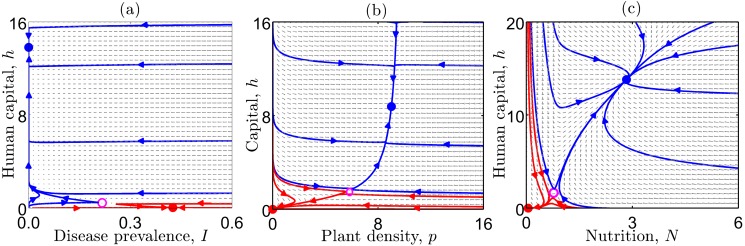
Disease and food systems exhibit bistability. Phase plots of (a) human capital against disease prevalence, (b) capital against plant density, and (c) human capital against nutrition showing two stable equilibria (solid circles), and one unstable equilibrium (open circle) in between. Sample trajectories that converge to the good equilibrium (solid blue circle) are denoted by blue lines, while sample trajectories that converge to the bad equilibrium (solid red circle) are denoted by red lines.

In the simple economic growth model presented in Figure 1c, capital accumulation exceeds depreciation at low levels of capital, allowing for further accumulation. The growth rate rises as capital rises from 0, but because of diminishing returns to capital, the system is ultimately forced to a single stable equilibrium. Coupling the model with the simple SIS model of infectious diseases creates a sigmoidal capital accumulation curve, similar to *ad hoc* models of poverty traps in the economics literature (Figure 2). The high disease burdens at low levels of income cause rates of human capital accumulation to be lower than capital depreciation, and therefore the population is stuck in poverty. The human capital accumulation curve rises rapidly in part of the state space, indicating that as human capital accumulates its rate of growth rises. This results from the effect of greater income on decreasing the basic reproductive ratio of disease: as income rises from low levels, the disease burden falls, which further increases rate of human capital accumulation. When human capital passes a threshold level, it accumulates faster than it depreciates, and a virtuous cycle ensues in the basin of attraction of the good equilibrium.

Note that through economic effects on the transmission and recovery rates in this multi-pathogen system, pathogens are facilitating each other. Increasing the number of pathogens in the system thus amplifies the positive feedback and expands the basin of attraction of the poverty trap as reflected in Figure 3d–f. This facilitation is ecological and economic (as opposed to immunological) in the sense that it occurs from antigenically distinct pathogens. It implies that the 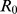 for all diseases falls as each pathogen is removed from the system. Total eradication can thus occur by eliminating a subset of the pathogens.

## Discussion

This Essay aims to make two conceptual points: (i) biophysical and biosocial processes often play fundamentally important roles in the structure of extreme poverty—these processes are often complex, based on nonlinear feedbacks; (ii) coupled models of economic growth and population ecology can provide a general framework for exploring the ecology of poverty and informing potential interventions.

As an example, we show how very simple infectious disease models can be integrated with economic growth models to give rise to poverty traps. When modeled independently, the simplest traditional models of economic growth and of infectious diseases each necessarily converge to a single stable equilibrium and therefore do not, alone, explain persistent differences in observed outcomes in the world: economically better-off populations have long since escaped the Malthusian trap while a subpopulation remains mired in a low productivity, low income, high disease state. Similar to processes in endogenous growth theory, here positive feedback is driven by interactions between income and human capital. Unlike typical economic models, however, these feedbacks occur through explicit ecological pathways.

Our model framework shows that even in a parsimonious case, a system of coupled economic growth and epidemiological dynamics can change underlying equilibrium income and disease phenomena, generating multiple stable states within a reasonable range of parameters. These outcomes can change on the basis of the number of pathogens in the system. While the evidence of the effects of immunologically based interactions between infectious diseases is ambiguous [67]–[69], this model shows that the effect of economically mediated interactions is less so: degrading human capital undermines economic productivity, increases disease transmission, and slows recovery rates, allowing antigenically distinct syndemic pathogens to facilitate each other. By amplifying economic feedbacks, the parameter space of bistable outcomes expands with the number of pathogens in the system.

The framework is meant to be simple and general. Though the model is based on conventional structure and parameters, the outcomes are nevertheless drawn from a specific set of assumptions about the production function and the transmission method. These assumptions can be adapted depending on the specific system of interest. Any incorporation of other factors to this framework, such as food production, reproductive behavior, population density, and resource use, present a host of other considerations, creating more potential feedback. Bistability is only one specific qualitative result from a spectrum of potentially important and interesting implications of such coupled models.

The importance of this approach to modeling poverty traps is 2-fold. First, the model can directly inform our understanding of the structural relationship between health and economic growth. It presents candidate sources of nonlinearities that can be routinely considered in theoretical and statistical analysis. While models of poverty traps based on nonlinear relationships between capital and income have existed for decades, these nonlinearities are typically phenomenological, rather than mechanistic, and are rarely derived from a rigorous understanding of underlying biophysical and biosocial processes. Accordingly, these assumptions have been challenged in the literature [70],[71]. Our model here also relies on a set of assumptions about phenomenological relationships in the transmission and human capital equations. However, these are more testable, lower-level, assumptions about relationships for which there is substantial empirical support: income reduces disease transmission and increases the rate of recovery, while infection reduces the rate of human capital accumulation. The functional relationships that we assume are conservative: for example, it turns out that if the model did not assume diminishing returns of income on transmission, then the sigmoidal curvature of the production function would have been even more pronounced. We thus show that relatively simple assumptions can give rise to complex (nonlinear) outcomes.

The second consideration is more important, more subtle, and likely be less obvious to the economics community than to the life sciences: the model points to an array of prospective linkages between biophysical and economic dynamics, with infectious diseases themselves representing an example of complex ecological agents. The theoretical literature in community and population ecology present fundamentally important concepts to our understanding of the consequences of such dynamics. Some of the leading causes of morbidity and mortality of the poor are vector-borne and parasitic diseases. Many such pathogens spend much of their life cycles outside of the human host, in free-living stages or in other host species. They depend on competition, predation, and a range of complex trophic interactions [51],[72]. The integration of human economic agents into ecological communities compounds the potential for complex dynamics, nonlinearity, and multiple stable states. These concepts are directly applicable to the many ways in which the economic productivity of the poor is tied to living systems. The dynamics of agriculture, for example, are directly dependent on similar ecological feedbacks that influence soil microbiota, hydrology, and plant disease.

A salient difference between empirical work in economic growth and in population ecology is that the latter has a stronger tradition of primary field-based data collection and a focus on dynamical processes, whereas the former has tended to specialize on secondary data analysis and equilibrium properties. Following the tradition of economics, the model presented here also focuses on equilibrium outcomes, but the most interesting and relevant properties of these kinds of systems may be their dynamics, which can be studied in real time in response to policy interventions. Even simple models of infectious diseases, as with many biophysical systems, can exhibit highly nonlinear behavior across space and time, from periodic dynamics to chaos [46]. The effects of such dynamics on human capital accumulation among the poor is almost entirely unexplored. Modeling efforts should accordingly be integrated with field projects that are affecting health or agroecological conditions at the population scale.

The past decade has borne witness to a new era of investment in global health and economic development broadly connected to the Millennium Development Goals, which explicitly aim to reduce global extreme poverty by 50% [56]. An explicit goal of ministries of health of many developing countries is to provide health care as part of a broader strategy for economic development [73]. In Rwanda, for example, which is on track to meet most of its Millennium Development Goals by 2015, the mortality rate of children under 5 is one-third of what it was at the turn of the Millennium, and per capita income has nearly doubled [74]. These results are connected to rapid changes in the strength of the health care system across all relevant scales: from community-based health, to local facilities, to the performance of reference hospitals. The effect of such policies that create access to health care independently of household income would serve to decouple this system, and accordingly break cycles of poverty and disease. Such radical shifts in disease and economic conditions create a special opportunity for economists and other social and natural scientists to contribute to a more integrated understanding of these coupled dynamics.

## Supporting Information

Figure S1
**The standard epidemiological parameters of (a) transmission** 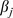 **and (b) recovery** 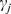 **are functions of income.** These are intuitive relationships, where income determines the specific rate that occurs between minimum and maximum levels that are biologically determined. (c) The rate of investment in human capital is proportional to the prevalence of disease.(TIF)

Figure S2 (**a) GDP per capita (income) versus per capita DALYs lost to infectious and parasitic diseases (disease burden), for developed countries (DC) and least developed countries (LDC).** (b) Natural log of income against natural log of disease burden for developed and developing countries.
(TIF)

Text S1 **Details of the economic model, the infectious disease model, the coupled economic-disease model, the coupled economic-agricultural model, the coupled economic-nutrition model, and calibration of the coupled economic-disease model (Figures S1 and S2).**
(PDF)
