# Supplementary figures and images for: Poverty, Disease, and the Ecology of Complex Systems
Source: PLoS Biol. 2014 Apr 1;12(4):e1001827. doi: 10.1371/journal.pbio.1001827 (PMC3972083; doi:10.1371/journal.pbio.1001827)

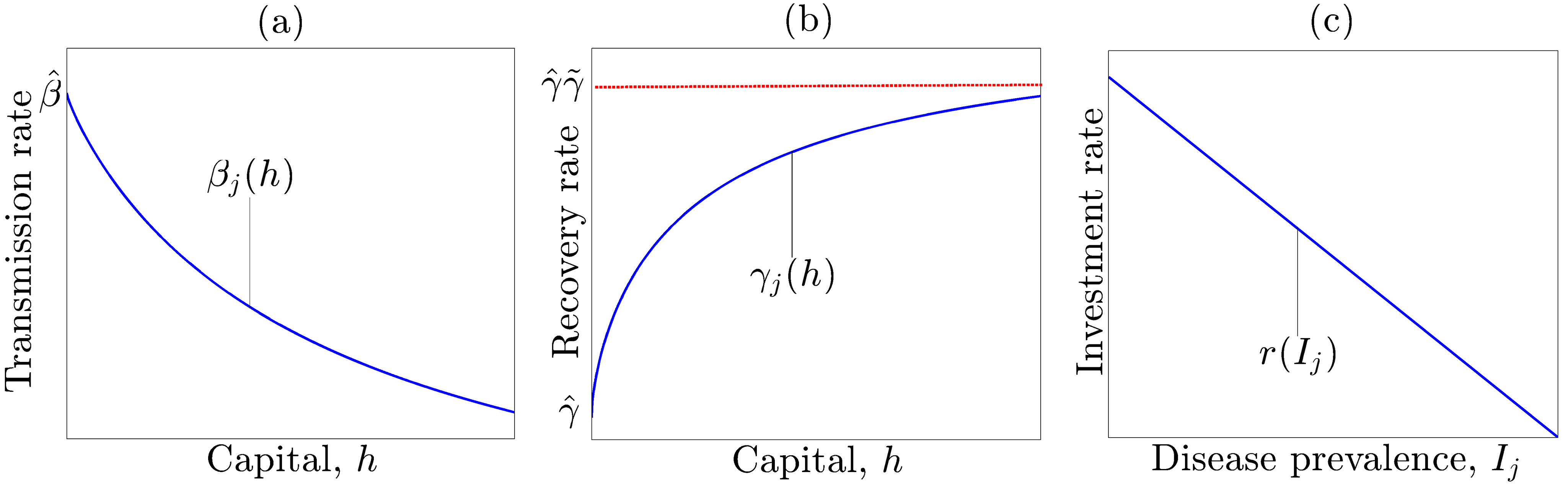

Supplement: Figure S1 — The standard epidemiological parameters of (a) transmission and (b) recovery are functions of income. These are intuitive relationships, where income determines the specific rate that occurs between minimum and maximum levels that are biologically determined. (c) The rate of investment in human capital is proportional to the prevalence of disease. (TIF) [file pbio.1001827.s001.tif]

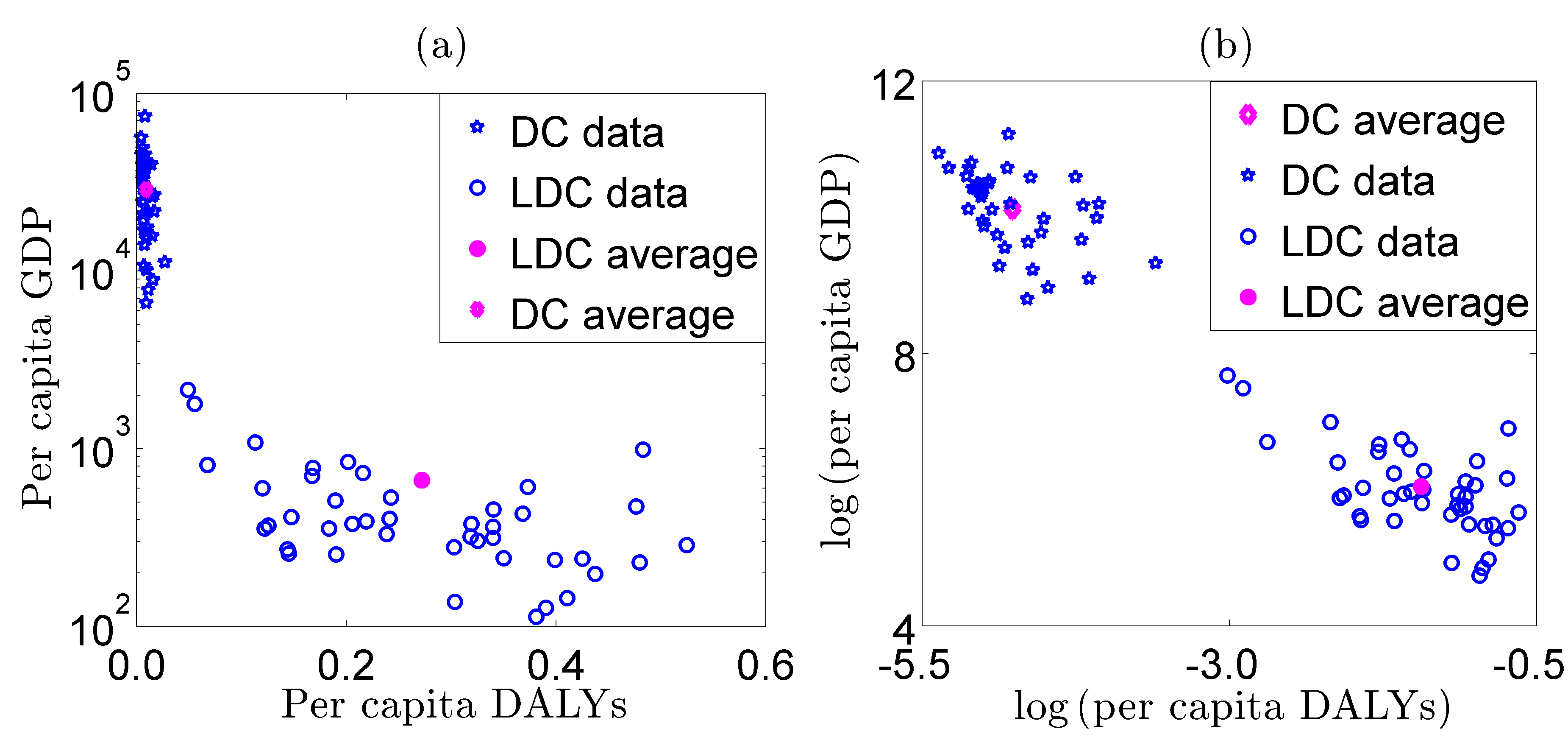

Supplement: Figure S2 — (a) GDP per capita (income) versus per capita DALYs lost to infectious and parasitic diseases (disease burden), for developed countries (DC) and least developed countries (LDC). (b) Natural log of income against natural log of disease burden for developed and developing countries. (TIF) [file pbio.1001827.s002.tif]
