# Supplementary material for: Poverty, Disease, and the Ecology of Complex Systems
Source: PLoS Biol. 2014 Apr 1;12(4):e1001827. doi: 10.1371/journal.pbio.1001827 (PMC3972083; doi:10.1371/journal.pbio.1001827)
Supplement: Text S1 — Details of the economic model, the infectious disease model, the coupled economic-disease model, the coupled economic-agricultural model, the coupled economic-nutrition model, and calibration of the coupled economic-disease model (Figures S1 and S2). (PDF) [file pbio.1001827.s003.pdf]

Poverty, disease and the ecology of complex systems: supporting information

Calistus N. Ngonghala, Mateusz M. Pluciński, Megan B. Murray, Paul E. Farmer, Christopher B Barrett, Donald C. Keenan, Matthew H. Bonds

Exogenous growth theory

Consider a constant-elasticity-of-substitution (CES) production function [1]:

$$Y(t) = (\alpha H(t)^{\rho} + (1-\alpha)(AL(t))^{\rho})^{1/\rho}, \tag{1}$$

where Y is total output (or income), t is time, H is the stock of capital, and L is the labor supply. The parameter $\alpha \in (0,1)$ is a distribution parameter, and $\sigma = 1/(1-\rho)$ is the elasticity of substitution between capital and technology-augmented labor. The CES function is among the most commonly employed production functions, and was among the original models presented by [2]. The CES is a generalized production function, which collapses to: 1) the Cobb-Douglas production function [3] as $\rho \to 0$ or as $\sigma \to 1$; 2) the linear production function as $\rho \to 1$ or as $\sigma \to \infty$; and 3) the Leontief production function [4] as $\rho \to \infty$ or as $\sigma \to 0$.

The standard neoclassical growth model [2] describes the rate of change of capital over time in accordance with the following equation:

$$\dot{H} = rY(t) - \delta_h H(t), \tag{2}$$

where r and δ_h are the respective rates of capital accumulation and depreciation. For notational convenience, we scale equations (1)-(2) by setting $y = Y/AL$ and $h = H/AL$, where y is defined as the effective output per unit of labor (or per capita income), such that:

$$y = (\alpha h^{\frac{\sigma-1}{\sigma}} + (1-\alpha))^{\frac{\sigma}{\sigma-1}}. \tag{3}$$

The intensive form of the economic growth model is:

$$\dot{h} = ry(t) - \delta h(t). \tag{4}$$

The population and technological growth rates are denoted by m and g respectively, so that $\delta = \delta_h + m + g$. Equation (4) is characterized by one globally stable equilibrium at h^*:

$$h^* = \left(\frac{\left(\frac{\delta}{r}\right)^{\frac{\sigma-1}{\sigma}} - \alpha}{1-\alpha} \right)^{\frac{\sigma}{\sigma-1}}. \tag{5}$$

The corresponding equilibrium value of y is

$$y^* = \left[\frac{1}{1-\alpha} - \frac{\alpha}{1-\alpha} \left(\frac{r}{\delta}\right)^{\frac{\sigma-1}{\sigma}} \right]^{-\frac{\sigma}{\sigma-1}}. \tag{6}$$

Infectious disease models

Equations (7) and (8) represent the most basic epidemic model:

$$\dot{S}_j = \gamma_j I_j - \beta_j I_j S_j, \tag{7}$$

$$\dot{I}_j = \beta_j I_j S_j - \gamma_j I_j, \tag{8}$$

where j refers to a specific pathogen (we incorporate multiple pathogens in the next section), and β and γ are the respective transmission and recovery rates. We assume a constant population; i.e., birth and death rates are equal and the infection is non-fatal. Dividing the susceptible and infectious populations by the total population yields proportions of susceptible and infectious individuals. For notational convenience, we preserve the same notation for proportions as for numbers (so that $S_j + I_j = 1$), where the system can be reduced to a single equation:

$$\dot{I}_j = \beta_j (1 - I_j) I_j - \gamma_j I_j. \tag{9}$$

The disease model given by Equation (9) admits two equilibrium solutions: a disease-free equilibrium solution $I_j^* = 0$, which exists and is globally and asymptotically stable when $R_{0j} = \frac{\beta_j}{\gamma_j} \leq 1$ and an endemic equilibrium solution $I_j^* = 1 - \frac{1}{R_{0j}}$, which exists and is globally and asymptotically stable when $R_{0j} > 1$. Stability of the disease-free equilibrium indicates that there is no disease the community, while stability of the endemic equilibrium indicates that the disease persists endemically in the community. The threshold quantity R_{0j} is the basic reproduction number of the jth disease. It represents the number of new infections introduced in a totally susceptible population by one infectious case during his/her infectious period.

Alternative common forms of disease models include the Susceptible-Infectious (SI) model, where infected individuals do not recover from infection; the Susceptible-Infectious-Removed (SIR) model, where infectious individuals recover by acquiring permanent immunity; the Susceptible-Infectious-Removed-Susceptible (SIRS) model, where the disease does not confer permanent immunity; the Susceptible-Exposed-Infectious-Removed (SEIR) model and Susceptible-Exposed-Infectious-Removed (SEIRS) model, where infected individuals go through an incubation period before becoming infectious.

Coupled disease-economic growth model

We couple the above system through two simple empirically-based mechanisms that form the basis for feedback between health and economic growth: 1) disease transmission and recovery are functions of income; and 2) human capital accumulation is a function of disease:

$$\beta_j(y) = \frac{\hat{\beta}\tilde{\beta}}{y(t)+\tilde{\beta}} \tag{10}$$

$$\gamma_j(y) = \frac{\hat{\gamma}\tilde{\gamma}(y(t)+1)}{y(t)+\tilde{\gamma}} \tag{11}$$

$$r(I_j) = r_c(1-I_j) \tag{12}$$

where $\hat{\beta} > 0, \tilde{\beta} > 0, \hat{\gamma} > 0$, and $\tilde{\gamma} > 0$ are exogenous parameters for the jth pathogen, with $\hat{\beta}$ and $\hat{\gamma}$ representing maximum transmission and minimum recovery rates. Equations (10) and (11) are based on previous analyses [5,6]. They assume that as income rises, the transmission rate falls and recovery rates rise. This corresponds to the fact that prevention and treatment of infectious diseases require economic resources, which is why poverty is a social determinant of disease [7, 8]. See Figure S1 for graphical illustrations.

Because human capital is one of the primary mechanisms through which the disease burden influences economic growth, our remaining model development focuses on this particular form of capital, which we still denote h. The parameter, r_c, can be regarded as per capita spending on education or training, which converts to human capital at a rate that depends on the health of the person. For example, there is substantial evidence that disease can cause significant decreases in school performance and attendance even for infections, such as hookworm, that have no outward signs of morbidity [9]. Because the most important effects of health on economic growth occur through the cumulative effects of all infections, we generalize equation (9) to represent n infectious diseases, which we assume do not directly impact each other through epidemiological mechanisms (i.e., the transmission and recovery

parameters for each pathogen are independent of each other). We define i as the probability that an individual has any infection: $i = 1 - \prod_{j=1}^{n} (1 - I_j)$. The corresponding endemic equilibrium is,

$$i^* = 1 - \prod_{j=1}^{n} 1/R_{0_j}, \tag{13}$$

which is the equilibrium proportion of the population that is infected by some pathogen. For simplicity, we assume that each of these diseases has the same transmission and recovery parameters; i.e., $\beta_j = \beta$, and $\gamma_j = \gamma$, $\forall\ j$ and therefore $I_j^* = I^*$. The equilibrium probability of being free of disease is then:

$$s^* = 1 - i^* = (1 - I^*)^n. \tag{14}$$

The rate of investment in human capital is $r = r_c(1 - I)^n$.

The dynamics of most infectious diseases occur on much shorter time scales than economic dynamics, which can occur over the course of generations. For the purposes of exploring the basic structure of this system, we assume the infectious disease dynamics quickly reach a quasi steady state. Combining equations (3), (4), (10), (11), (12), (13), and (14) yields a single equation for the dynamics of human capital in the presence of disease:

$$\dot{h} = r_c \left(\frac{\gamma(y)}{\beta(y)} \right)^n y - \delta h. \tag{15}$$

Model calibration

We use MATLAB and maximum likelihood estimation to calibrate the parameters, β_0, β_1, γ_0, γ_1, r_c, δ, and n by fitting the model to health and income statistics. We treat n as a free parameter to represent the "effective" number of diseases, given that many different kinds of infections are aggregated in the data (such as diarrheal diseases and respiratory infections). Where possible, we rely on existing parameter estimates found in the literature; the economic parameters, $\alpha = 0.5$ and $\sigma = 1.4$, are drawn from [10, 11].

In the tradition of [5] we focus on the 83 countries that are either considered "advanced" (DC) or "least developed" (LDC) by the International Monetary Fund, and so do not include countries that are identified as being in transition. These data are presented in Figure S2. We approximate the steady-state infectious disease burden with the per capita DALYs attributable to infectious and parasitic diseases from the most recent W.H.O. Global Burden of Disease Database [12]. The parameters are estimated by finding the set of parameters that minimizes the squared

distance between the expected and observed values of I and y. The estimated parameters are $\beta_0 = 9.1968, \beta_1 = 11.7348, \gamma_0 = 4.1992, \gamma_1 = 21.0637, r_c = 0.3728, \delta = 0.1276, n = 6.2035, \alpha = 0.5, \sigma = 1.4$. Notice that these values are consistent with intuition and existing literature. The maximum rate of investment in development (or education) is $r_c = 0.37$, which can drop to half of that if a child is often sick. The rate of capital depreciation, $\delta = 0.1276$, falls well within the range of prevailing estimates for research and development capital [13–15]. The estimated number of pathogens is about $n = 6$, which could broadly capture the most essential infections in a system.

Models for Box 2

Agriculture model

The simple agriculture model is based off the plant growth framework of [16, 17]. The plant growth model follows the traditional logistic growth-type pattern with adjustments for harvesting and/or other processes that reduce plant density such as grazing by herbivores. Suppose p denotes plant density and h denotes human capital, then the coupled model system is given by

$$\dot{p} = r_p p \left(1 - \frac{p}{K}\right) h^{\sigma} - \epsilon p, \tag{16}$$

$$\dot{h} = r_c \left(\frac{p}{p_0 + p}\right) h^{\sigma} - \delta h, \tag{17}$$

where r_p is the intrinsic growth rate of plants, K is the environmental carrying capacity, ϵ yield or harvesting rate, p_0 is a scaling or half saturation constant, and the other parameters are as described in Equations (3) and (4); $r_c = 0.3728, \delta = 0.1276, \sigma = 0.85, r_p = 0.4, \epsilon = 0.25, K = 10$, and $p_0 = 10$

Nutrition model

Nutrition uptake can be modeled as a classic ecological consumer-resource system, such as has been used to represent rates of feeding and energy transfer [18–23]. We use standard saturation functional forms and the neoclassical growth model formulation (4) with the CES production function (3) to build a coupled system of nutrition and human capital. Accumulation of nutrition occurs through a ratio-dependent functional response [21, 24, 25] term with maximum accumulation constant λ, and nutrition uptake or consumption is through a linear term ϵ. The

nutrition model is coupled to the human capital model through a Holling Type III functional response [18–20] with half-saturation constant N_0 in the investment rate in human capital, while human capital is coupled to the nutrition model through an income ratio-dependent term in the nutrition-accumulation rate. A Holling Type III functional response is a sigmoidal-shaped function used in studying a range of biological processes, including consumer-resource systems in ecology. Such functional responses exhibit downward concavity at low resource density. The coupled model is described by the following system of equations:

$$\dot{h} = r_c \frac{N^m}{N_0 + N^m} y - \delta h, \tag{18}$$

$$\dot{N} = \lambda \frac{y}{y + N} N - \epsilon N, \tag{19}$$

where h, y, r_c and δ are as in Equations (3)-(4), N is nutrition and m is a shape constant; $r_c = 0.3728, \delta = 0.1276, \lambda = 0.4, \epsilon = 0.25, f_0 = 0.1$.

For the coupled disease-economic model, we assumed that disease dynamics attain a quasi-steady state quickly since the dynamics of some diseases occur on faster time scale (days, weeks, months or a few years) compared to economic growth dynamics, which might span one or more generations. This allowed us to reduce the coupled system of ordinary differential equations into a single equation. Because the relationship between renewable resources and human capital may occur on similar economic time scales, we work with the two coupled equations in each case.

References

1. Arrow KJ, Chenery HB, Minhas BS, Solow RM (1961) Capital-labor substitution and economic efficiency. The Review of Economics and Statistics 43: 225–250.

2. Solow R (1956) A contribution to the theory of economic growth. The Quarterly Journal of Economics 70 (1): 65–94.

3. Cobb CW, Douglas PH (1928) A theory of production. The American Economic Review 18: 139–165.

4. Leontief WW (1941) Structure of american economy, 1919-1929 .

5. Bonds M, Keenan D, Rohani P, Sachs J (2010) Poverty trap formed by the ecology of infectious diseases. Proceedings of the Royal Society B: Biological Sciences 277: 1185–1192.

6. Pluciński MM, Ngonghala CN, Getz WM, Bonds MH (2013) Clusters of poverty and disease emerge from feedbacks on an epidemiological network. Journal of The Royal Society Interface 10.

7. Stevens P (2004) 4 diseases of poverty and the 10/90 gap. International Policy Network .

8. UN Panel Report (2005) Poverty, infectious disease, and environmental degradation as threats to collective security. Population and development review 31 (3): 595–600.

9. Miguel E, Kremer M (2004) Identifying impact on education and health in the presence of treatment externalities. Econometrica 72: 159–217.

10. Barro R, Sala-i Martin X (1999) Economic growth. Cambridge, MA: MIT Press.

11. Masanjala W, Papageorgiou C (2004) The solow model with ces technology: nonlinearities and parameter heterogeneity. Journal of Applied Econometrics 19: 171–201.

12. Lopez AD, Mather CD, Ezzati M, Jamison DT, Murray CJL (2006) Global burden of disease and risk factors. New York, NY: Oxford University Press.

13. Pakes A, Schankerman M (1986) Estimates of the value of patent rights in European countries during the post-1950 period. Economic Journal 96: 1052–1076.

14. Nadiri MI, Prucha IR (1996) Estimation of the depreciation rate of physical and R&D capital in the U.S. total manufacturing sector. Economic Inquiry 34 (1): 43–56.

15. Bernstein JI, Mamuneas T (2006) R&D depreciation, stocks, user costs and productivity growth for U.S. R&D intensive industries. Structural Change and Economic Dynamics, 17(1): 17 (1): 70–98.

16. Rietkerk M, Van de Koppel J (1997) Alternate stable states and threshold effects in semi-arid grazing systems. Oikos : 69–76.

17. van de Koppel J, Rietkerk M, Weissing FJ (1997) Catastrophic vegetation shifts and soil degradation in terrestrial grazing systems. Trends in Ecology & Evolution 12: 352–356.

18. Holling CS (1959a) The components of predation as revealed by a study of small mammal predation of the European pine sawfly. The Canadian Entomologist 91: 385–398.

19. Holling CS (1959b) Some characteristics of simple types of predation and parasitism. The Canadian Entomologist 91: 385–398.

20. Dawes J, Souza M (2013) A derivation of holling's type i, {II} and {III} functional responses in predator-prey systems. Journal of Theoretical Biology 327: 11 - 22.

21. DeAngelis DL, Goldstein R, O'neill R (1975) A model for tropic interaction. Ecology : 881–892.

22. Ryrie SC, Prentice IC (2011) Herbivores enable plant survival under nutrient limited conditions in a model grazing system. Ecological Modelling 222: 381–397.

23. Bonachela JA, Raghib M, Levin SA (2011) Dynamic model of flexible phytoplankton nutrient uptake. Proceedings of the National Academy of Sciences 108: 20633–20638.

24. Arditi R, Ginzburg LR (1989) Coupling in predator-prey dynamics: ratio-dependence. Journal of Theoretical Biology 139: 311–326.

25. Akçakaya HR, Arditi R, Ginzburg LR (1995) Ratio-dependent predation: an abstraction that works. Ecology : 995–1004.

Figure captions

Figure S1: The standard epidemiological parameters of a) transmission β_j and b) recovery γ_j are functions of income. These are intuitive relationships, where income determines the specific rate that occurs between minimum and maximum levels that are biologically determined. c) The rate of investment in human capital is proportional to the prevalence of disease.

Figure S2: (a) GDP per capita (income) versus per capita DALYs lost to infectious and parasitic diseases (Disease Burden), for developed countries (DC) and least developed countries (LDC). (b) Natural log of income against natural log of disease burden for developed and developing countries.
